# Supplementary material for: High G2M Pathway Score Pancreatic Cancer is Associated with Worse Survival, Particularly after Margin-Positive (R1 or R2) Resection
Source: Cancers (Basel). 2020 Oct 6;12(10):2871. doi: 10.3390/cancers12102871 (PMC7599494; doi:10.3390/cancers12102871)
Supplement: Supplementary file 1 [file cancers-12-02871-s001.pdf]

# Supplementary Material: High G2M Pathway Score Pancreatic Cancer is Associated with Worse Survival, Particularly after Margin-Positive (R1 or R2) Resection

**Masanori Oshi, Stephanie Newman, Yoshihisa Tokumaru, Li Yan, Ryusei Matsuyama, Itaru Endo, Matthew H.G. Katz and Kazuaki Takabe**

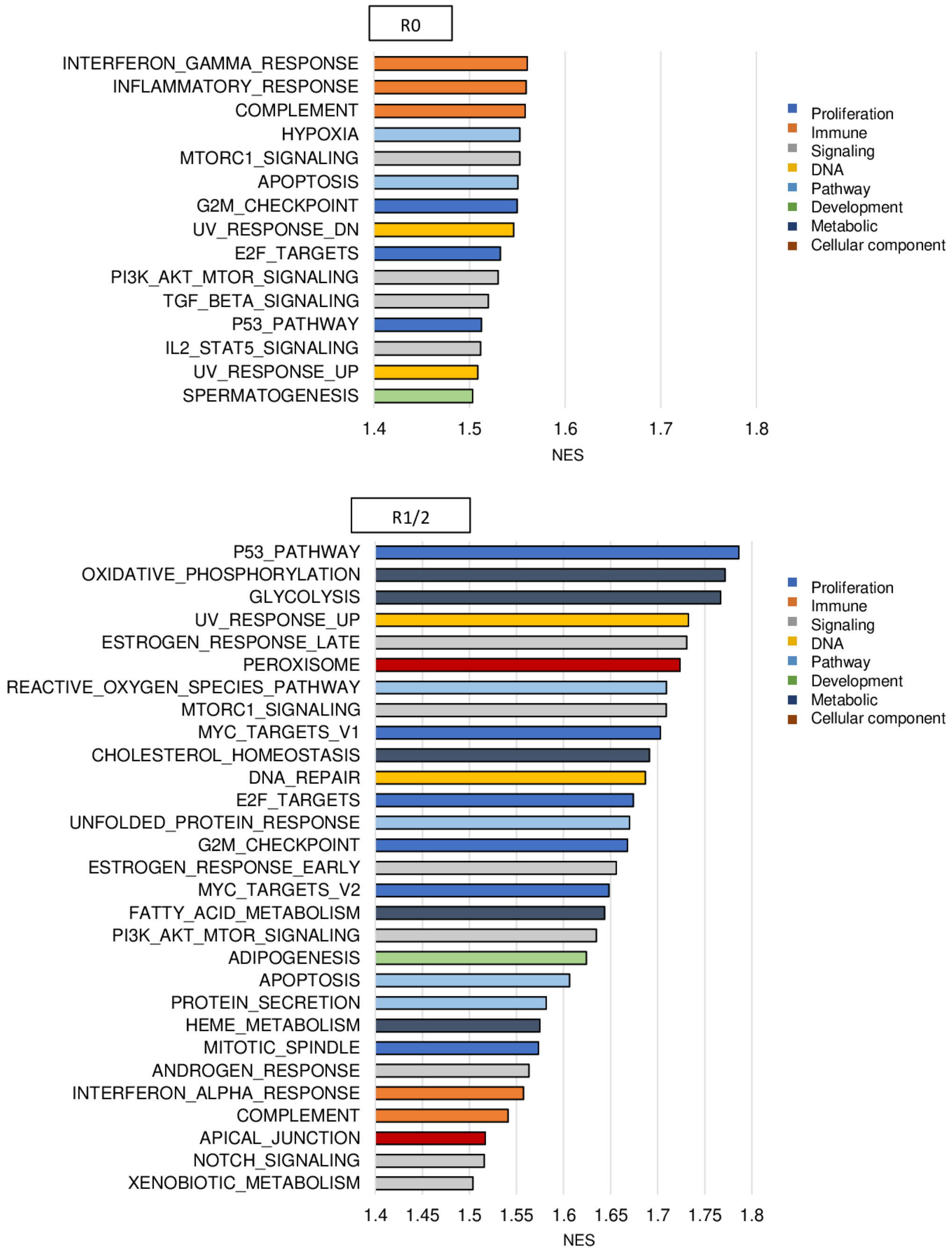


**Figure S1.** Hallmark gene sets significantly enriched in G2M pathway score high tumors in R0 or R1/2 groups in the TCGA cohort. Bar plots of normalized enrichment score [NES] of each Hallmark gene sets for which significant enrichment (NES > 1.5 and false discovery rate [FDR] < 0.25) was observed in G2M high score compared to low score tumors in R0 and R1/2 groups.

**Table S1.** Gene name and Symbol of the G2M target pathway.

| Gene Symbol | Gene Title |
|---|---|
| ABL1 | ABL proto-oncogene 1, non-receptor tyrosine kinase |
| AC027237.1 | kinesin family member 23 |
| AC091021.1 | SS18 subunit of BAF chromatin remodeling complex |
| AMD1 | adenosylmethionine decarboxylase 1 |
| ARID4A | AT-rich interaction domain 4A |
| ATF5 | activating transcription factor 5 |
| ATRX | ATRX chromatin remodeler |
| AURKA | aurora kinase A |
| AURKB | aurora kinase B |
| BARD1 | BRCA1 associated RING domain 1 |
| BCL3 | BCL3 transcription coactivator |
| BIRC5 | baculoviral IAP repeat containing 5 |
| BRCA2 | BRCA2 DNA repair associated |
| BUB1 | BUB1 mitotic checkpoint serine/threonine kinase |
| BUB3 | BUB3 mitotic checkpoint protein |
| CASP8AP2 | caspase 8 associated protein 2 |
| CBX1 | chromobox 1 |
| CCNA2 | cyclin A2 |
| CCNB2 | cyclin B2 |
| CCND1 | cyclin D1 |
| CCNF | cyclin F |
| CCNT1 | cyclin T1 |
| CDC20 | cell division cycle 20 |
| CDC25A | cell division cycle 25A |
| CDC25B | cell division cycle 25B |
| CDC27 | cell division cycle 27 |
| CDC45 | cell division cycle 45 |
| CDC6 | cell division cycle 6 |
| CDC7 | cell division cycle 7 |
| CDK1 | cyclin dependent kinase 1 |
| CDK4 | cyclin dependent kinase 4 |
| CDKN1B | cyclin dependent kinase inhibitor 1B |
| CDKN2C | cyclin dependent kinase inhibitor 2C |
| CDKN3 | cyclin dependent kinase inhibitor 3 |
| CENPA | centromere protein A |
| CENPE | centromere protein E |
| CENPF | centromere protein F |
| CHAF1A | chromatin assembly factor 1 subunit A |
| CHEK1 | STT3 oligosaccharyltransferase complex catalytic subunit A |
| CHMP1A | charged multivesicular body protein 1A |
| CKS1B | CDC28 protein kinase regulatory subunit 1B |
| CKS2 | CDC28 protein kinase regulatory subunit 2 |
| CTCF | CCCTC-binding factor |
| CUL1 | cullin 1 |
| CUL3 | cullin 3 |
| CUL4A | cullin 4A |
| CUL5 | cullin 5 |
| DBF4 | DBF4 zinc finger |
| DDX39A | DExD-box helicase 39A |
| DKC1 | dyskerin pseudouridine synthase 1 |
| DMD | dystrophin |
| DR1 | down-regulator of transcription 1 |
| DTYMK | deoxythymidylate kinase |
| E2F1 | E2F transcription factor 1 |
| E2F2 | E2F transcription factor 2 |

| | |
|---|---|
| E2F3 | E2F transcription factor 3 |
| E2F4 | E2F transcription factor 4 |
| EFNA5 | ephrin A5 |
| EGF | epidermal growth factor |
| ESPL1 | extra spindle pole bodies like 1, separase |
| EWSR1 | EWS RNA binding protein 1 |
| EXO1 | exonuclease 1 |
| EZH2 | enhancer of zeste 2 polycomb repressive complex 2 subunit |
| FANCC | FA complementation group C |
| FBXO5 | F-box protein 5 |
| FOXN3 | forkhead box N3 |
| G3BP1 | G3BP stress granule assembly factor 1 |
| GINS2 | GINS complex subunit 2 |
| GSPT1 | G1 to S phase transition 1 |
| H2AFV | H2A histone family member V |
| H2AFX | H2A histone family member X |
| H2AFZ | H2A histone family member Z |
| HIF1A | hypoxia inducible factor 1 subunit alpha |
| HIRA | histone cell cycle regulator |
| HIST1H2BK | histone H2B type F-S-like |
| HMGA1 | high mobility group AT-hook 1 |
| HMGB3 | high mobility group box 3 |
| HMGN2 | high mobility group nucleosomal binding domain 2 |
| HMMR | hyaluronan mediated motility receptor |
| HNRNPD | heterogeneous nuclear ribonucleoprotein D |
| HNRNPU | heterogeneous nuclear ribonucleoprotein U |
| HOXC10 | homeobox C10 |
| HSPA8 | Heat shock 70 kDa protein 8 |
| HUS1 | HUS1 checkpoint clamp component |
| ILF3 | interleukin enhancer binding factor 3 |
| INCENP | inner centromere protein |
| JPT1 | Jupiter microtubule associated homolog 1 |
| KATNA1 | katanin catalytic subunit A1 |
| KIF11 | kinesin family member 11 |
| KIF15 | kinesin family member 15 |
| KIF20B | kinesin family member 20B |
| KIF22 | kinesin family member 22 |
| KIF2C | kinesin family member 2C |
| KIF4A | kinesin family member 4A |
| KIF5B | kinesin family member 5B |
| KMT5A | lysine methyltransferase 5A |
| KNL1 | kinetochore scaffold 1 |
| KPNA2 | karyopherin subunit alpha 2 |
| KPNB1 | karyopherin subunit beta 1 |
| LBR | Lamin B receptor |
| LIG3 | DNA ligase 3 |
| LMNB1 | lamin B1 |
| MAD2L1 | mitotic arrest deficient 2 like 1 |
| MAP3K20 | mitogen-activated protein kinase kinase kinase 20 |
| MAPK14 | mitogen-activated protein kinase 14 |
| MARCKS | myristoylated alanine rich protein kinase C substrate |
| MCM2 | minichromosome maintenance complex component 2 |
| MCM3 | minichromosome maintenance complex component 3 |
| MCM5 | minichromosome maintenance complex component 5 |
| MCM6 | minichromosome maintenance complex component 6 |
| MEIS1 | Meis homeobox 1 |
| MEIS2 | Meis homeobox 2 |
| MKI67 | marker of proliferation Ki-67 |

| | |
|---|---|
| MNAT1 | MNAT1 component of CDK activating kinase |
| MT2A | metallothionein 2A |
| MTF2 | metal response element binding transcription factor 2 |
| MYBL2 | MYB proto-oncogene like 2 |
| MYC | MYC proto-oncogene, bHLH transcription factor |
| NASP | nuclear autoantigenic sperm protein |
| NCL | nucleolin |
| NDC80 | NDC80 kinetochore complex component |
| NEK2 | NIMA related kinase 2 |
| NOLC1 | nucleolar and coiled-body phosphoprotein 1 |
| NOTCH2 | notch receptor 2 |
| NSD2 | nuclear receptor binding SET domain protein 2 |
| NUMA1 | nuclear mitotic apparatus protein 1 |
| NUP50 | nucleoporin 50 |
| NUP98 | nucleoporin 98 |
| NUSAP1 | nucleolar and spindle associated protein 1 |
| ODC1 | ornithine decarboxylase 1 |
| ODF2 | outer dense fiber of sperm tails 2 |
| ORC5 | origin recognition complex subunit 5 |
| ORC6 | origin recognition complex subunit 6 |
| PAFAH1B1 | platelet activating factor acetylhydrolase 1b regulatory subunit 1 |
| PBK | PDZ binding kinase |
| PDS5B | PDS5 cohesin associated factor B |
| PLK1 | polo like kinase 1 |
| PLK4 | polo like kinase 4 |
| PML | promyelocytic leukemia |
| POLA2 | DNA polymerase alpha 2, accessory subunit |
| POLE | DNA polymerase epsilon, catalytic subunit |
| POLQ | DNA polymerase theta |
| PRC1 | PPARG related coactivator 1 |
| PRIM2 | DNA primase subunit 2 |
| PRMT5 | protein arginine methyltransferase 5 |
| PRPF4B | pre-mRNA processing factor 4B |
| PTTG1 | PTTG1 regulator of sister chromatid separation, securin |
| PTTG3P | Pituitary tumor-transforming 3, pseudogene |
| PURA | purine rich element binding protein A |
| RACGAP1 | Rac GTPase activating protein 1 |
| RAD21 | RAD21 cohesin complex component |
| RAD23B | RAD23 homolog B, nucleotide excision repair protein |
| RAD54L | RAD54 like |
| RASAL2 | RAS protein activator like 2 |
| RBL1 | RB transcriptional corepressor like 1 |
| RBM14 | RNA binding motif protein 14 |
| RPA2 | replication protein A2 |
| RPS6KA5 | ribosomal protein S6 kinase A5 |
| SAP30 | Sin3A associated protein 30 |
| SFPQ | splicing factor proline and glutamine rich |
| SLC12A2 | solute carrier family 12 member 2 |
| SLC38A1 | solute carrier family 38 member 1 |
| SLC7A1 | solute carrier family 7 member 1 |
| SLC7A5 | solute carrier family 7 member 5 |
| SMAD3 | SMAD family member 3 |
| SMARCC1 | SWI/SNF related, matrix associated, actin dependent regulator of chromatin subfamily c member 1 |
| SMC1A | structural maintenance of chromosomes 1A |
| SMC2 | structural maintenance of chromosomes 2 |
| SMC4 | structural maintenance of chromosomes 4 |
| SNRPD1 | small nuclear ribonucleoprotein D1 polypeptide |
| SQLE | squalene epoxidase |

| | |
|---|---|
| SRSF1 | serine and arginine rich splicing factor 1 |
| SRSF10 | serine and arginine rich splicing factor 10 |
| SRSF2 | serine and arginine rich splicing factor 2 |
| STAG1 | stromal antigen 1 |
| STIL | STIL centriolar assembly protein |
| STMN1 | stathmin 1 |
| SUV39H1 | suppressor of variegation 3-9 homolog 1 |
| SYNCRIP | synaptotagmin binding cytoplasmic RNA interacting protein |
| TACC3 | transforming acidic coiled-coil containing protein 3 |
| TENT4A | terminal nucleotidyltransferase 4A |
| TFDP1 | transcription factor Dp-1 |
| TGFB1 | transforming growth factor beta 1 |
| TLE3 | TLE family member 3, transcriptional corepressor |
| TMPO | thymopoietin |
| TNPO2 | transportin 2 |
| TOP1 | DNA topoisomerase I |
| TOP2A | DNA topoisomerase II alpha |
| TPX2 | TPX2 microtubule nucleation factor |
| TRA2B | transformer 2 beta homolog |
| TRAIP | TRAF interacting protein |
| TROAP | trophinin associated protein |
| TTK | TTK protein kinase |
| UBE2C | ubiquitin conjugating enzyme E2 C |
| UBE2S | ubiquitin conjugating enzyme E2 S |
| UCK2 | uridine-cytidine kinase 2 |
| UPF1 | UPF1 RNA helicase and ATPase |
| WRN | Werner syndrome RecQ like helicase |
| XPO1 | exportin 1 |
| YTHDC1 | YTH domain containing 1 |

**Table S2.** Overlaps genes of G2M target pathway with Hallmark gene sets.

| Gene Set Name | Total Genes | Genes in Overlap |
|---|---|---|
| HALLMARK_G2M_CHECKPOINT | 200 | 200 |
| HALLMARK_E2F_TARGETS | 200 | 73 (36.5%) |
| HALLMARK_MITOTIC_SPINDLE | 199 | 39 (19.6%) |
| HALLMARK_MYC_TARGETS_V1 | 200 | 29 (14.5%) |
| HALLMARK_NOTCH_SIGNALING | 32 | 4 (12.5%) |
| HALLMARK_MYC_TARGETS_V2 | 58 | 6 (10.3%) |
| HALLMARK_SPERMATOGENESIS | 135 | 11 (8.1%) |
| HALLMARK_UV_RESPONSE_DN | 144 | 9 (6.3%) |
| HALLMARK_HEDGEHOG_SIGNALING | 36 | 2 (5.6%) |
| HALLMARK_UNFOLDED_PROTEIN_RESPONSE | 113 | 6 (5.3%) |
| HALLMARK_WNT_BETA_CATENIN_SIGNALING | 42 | 2 (4.8%) |
| HALLMARK_MTORC1_SIGNALING | 200 | 9 (4.5%) |
| HALLMARK_ESTROGEN_RESPONSE_LATE | 200 | 8 (4.0%) |
| HALLMARK_PI3K_AKT_MTOR_SIGNALING | 105 | 4 (3.8%) |
| HALLMARK_TGF_BETA_SIGNALING | 54 | 2 (3.7%) |
| HALLMARK_CHOLESTEROL_HOMEOSTASIS | 74 | 2 (2.7%) |
| HALLMARK_APOPTOSIS | 161 | 4 (2.5%) |
| HALLMARK_APICAL_SURFACE | 44 | 1 (2.3%) |
| HALLMARK_IL2_STAT5_SIGNALING | 200 | 4 (2.0%) |
| HALLMARK_FATTY_ACID_METABOLISM | 158 | 3 (1.9%) |
| HALLMARK_PEROXISOME | 104 | 2 (1.9%) |
| HALLMARK_UV_RESPONSE_UP | 158 | 3 (1.9%) |
| HALLMARK_ALLOGRAFT_REJECTION | 200 | 3 (1.5%) |
| HALLMARK_ESTROGEN_RESPONSE_EARLY | 200 | 3 (1.5%) |
| HALLMARK_HEME_METABOLISM | 200 | 3 (1.5%) |
| HALLMARK_HYPOXIA | 200 | 3 (1.5%) |
| HALLMARK_INFLAMMATORY_RESPONSE | 200 | 3 (1.5%) |
| HALLMARK_INTERFERON_GAMMA_RESPONSE | 200 | 3 (1.5%) |
| HALLMARK_DNA_REPAIR | 150 | 2 (1.3%) |
| HALLMARK_IL6_JAK_STAT3_SIGNALING | 87 | 1 (1.1%) |
| HALLMARK_ANDROGEN_RESPONSE | 100 | 1 (1.0%) |
| HALLMARK_MYOGENESIS | 200 | 2 (1.0%) |
| HALLMARK_APICAL_JUNCTION | 200 | 2 (1.0%) |
| HALLMARK_EPITHELIAL_MESENCHYMAL_TRANSITION | 200 | 2 (1.0%) |
| HALLMARK_KRAS_SIGNALING_DN | 200 | 2 (1.0%) |
| HALLMARK_ADIPOGENESIS | 200 | 1 (0.5%) |
| HALLMARK_P53_PATHWAY | 200 | 1 (0.5%) |
| HALLMARK_XENOBIOTIC_METABOLISM | 200 | 1 (0.5%) |
| HALLMARK_ANGIOGENESIS | 36 | 0 |
| HALLMARK_BILE_ACID_METABOLISM | 112 | 0 |
| HALLMARK_COAGULATION | 138 | 0 |
| HALLMARK_COMPLEMENT | 200 | 0 |
| HALLMARK_INTERFERON_ALPHA_RESPONSE | 97 | 0 |
| HALLMARK_KRAS_SIGNALING_UP | 200 | 0 |
| HALLMARK_OXIDATIVE_PHOSPHORYLATION | 200 | 0 |
| HALLMARK_PANCREAS_BETA_CELLS | 40 | 0 |
| HALLMARK_PROTEIN_SECRETION | 96 | 0 |
| HALLMARK REACTIVE OXYGEN SPECIES PATHWAY | 49 | 0 |
